# Emotion Classification in Japanese Cancer Survivor Interview Narratives Using Sentiment Polarity and Plutchik Emotion Frameworks: Model Development and Evaluation Study

**DOI:** 10.2196/94826

**Published:** 2026-06-30

**Authors:** Soma Hisamura, Satoshi Watabe, Hayato Kizaki, Shungo Imai, Kyoko Sayama, Toru Kishida, Natsumi Fukuoka, Shuntaro Yada, Eiji Aramaki, Satoko Hori

**Affiliations:** 1 Division of Drug Informatics, Keio University Faculty of Pharmacy, 1-5-30 Shibakoen, Minato-ku, Tokyo, 105-8512, Japan, 81 3-5400-2650; 2 Cancer Note, Nonprofit Organization, Tokyo, Japan; 3 Faculty of Library, Information and Media Science, University of Tsukuba, Ibaraki, Japan; 4 Division of Information Science, Graduate School of Science and Technology, Nara Institute of Science and Technology, Nara, Japan

**Keywords:** cancer survivorship, natural language processing, emotion classification, patient narratives, psychosocial support, sentiment analysis

## Abstract

**Background:**

Cancer survivors often experience complex and coexisting emotions throughout diagnosis, treatment, and posttreatment life. Emotion classification of patient narratives may help in understanding survivorship experiences; however, evidence remains limited for multidimensional classification using cancer survivor interview narratives.

**Objective:**

This study aimed to develop and evaluate natural language processing–based emotion classification models using Japanese cancer survivor interview narratives and to examine whether polarity and multidimensional emotion labels provide complementary perspectives.

**Methods:**

We analyzed verbatim transcripts from 15 cancer survivor interviews published by the Cancer Note, Nonprofit Organization. Survivor utterances were extracted, noninformative conversational elements were removed, texts were segmented at Japanese punctuation marks, and 5 consecutive sentences were grouped into 1 chunk. Two annotators labeled 1998 text chunks with 3-class sentiment polarity labels (positive, neutral, or negative) and multilabel Plutchik 8-emotion labels (joy, trust, fear, surprise, sadness, disgust, anger, and anticipation). Japanese BERT (Bidirectional Encoder Representations from Transformers) and LUKE (Language Understanding with Knowledge-based Embeddings) were fine-tuned to build a multiclass polarity classifier and a multilabel 8-emotion classifier. Performance was evaluated using precision, recall, *F*_1_-score, macroaveraged metrics, Micro-*F*_1_ for polarity, and Hamming loss for multilabel classification. For comparison, the same architectures were fine-tuned on WRIME (writers’ and readers’ intensities of emotion for their estimation), a Japanese social media emotion dataset, and evaluated on Cancer Note texts as a domain-transfer analysis. The 95% CIs were estimated using bootstrap resampling with 1000 iterations.

**Results:**

Neutral was the most frequent polarity label, trust was the most frequent 8-emotion label, and anger was the least frequent emotion label. Label distributions were imbalanced, with most-to-least frequency ratios of 3.47 for polarity and 8.10 for 8-emotion labels. In the 3-class sentiment polarity task, interview-trained models outperformed WRIME-trained transfer models. Interview Text-BERT achieved the highest micro-*F*_1_ of 0.696 (95% CI 0.676‐0.716), whereas Interview Text-LUKE achieved the highest macro-*F*_1_ of 0.660 (95% CI 0.639‐0.682). In the 8-emotion multilabel task, Interview Text-LUKE achieved the highest macro-*F*_1_ of 0.427 (95% CI 0.398‐0.453) and the lowest Hamming loss of 0.078 (95% CI 0.073‐0.082). WRIME-trained transfer models showed lower performance, particularly in the 8-emotion task. Sadness and trust co-occurred most frequently, suggesting that positive and negative emotional elements may coexist in the same narratives.

**Conclusions:**

This exploratory study suggests the feasibility of domain-specific emotion classification for Japanese cancer survivor interview narratives. Models fine-tuned on target-domain narratives generally outperformed WRIME-trained transfer models, although the best architecture differed by task and metric. Polarity labels and Plutchik 8-emotion labels provided complementary perspectives on complex and coexisting emotions in survivorship narratives. However, performance for rare emotions remained limited, and the models should be regarded as preliminary research tools rather than clinically actionable systems. Larger, more diverse, prospectively or externally validated datasets, imbalance-aware methods, and user-centered evaluation are needed before clinical translation.

## Introduction

Patients with cancer experience a diverse and complex range of emotions throughout their journey—diagnosis, treatment, and follow-up care. These emotional experiences profoundly influence their decision-making and psychological well-being [1,2]. Emotions significantly shape patients’ decisions, as highlighted by Treffers and Putora [3], emphasizing the importance of understanding these dynamics for short-term mental care and improved long-term clinical outcomes [4-6].

The emerging “social model” of health suggests that mental and emotional well-being is influenced not only by individual experiences but also by social and contextual factors, such as access to health care resources and support networks [7,8]. This broader perspective is essential, as patients with cancer frequently encounter emotional challenges shaped by both personal and environmental factors.

Previous research has primarily classified emotions using sentiment polarity-based methods, categorizing them as positive, neutral, or negative. Recent studies have applied natural language processing (NLP) and sentiment analysis to cancer-related social media posts, blogs, clinical records, and patient-generated texts, suggesting that computational methods can help characterize emotional expressions and psychosocial concerns in oncology-related narratives. However, most prior work has focused on polarity-based sentiment classification or specific online text sources, and fewer studies have examined multidimensional emotion classification using interview narratives from cancer survivors. In particular, limited evidence exists on whether models trained on general emotion datasets can transfer to retrospective survivorship interviews or whether domain-specific narrative data are needed to capture coexisting and context-dependent emotions. While these approaches capture general psychological patterns, they often overlook the nuanced and multifaceted emotional experiences of patients [9]. For instance, individuals may simultaneously experience positive and negative emotions [10,11] or undergo rapid and subtle emotional shifts over short periods [12]. Multidimensional models, such as those proposed by Plutchik [13] and Ekman [14], offer a more detailed framework for analyzing emotions. Integrating sentiment polarity analysis with multidimensional emotion models may enable a deeper understanding of patients’ emotional states, accounting for personal, social, and contextual influences, as emphasized in the social model.

This study analyzed interview narratives of cancer survivors to better understand these complex emotions. These narratives, expressed in patients’ own words and inherently retrospective, provide direct insights into their feelings [15,16]. Analyzing such narratives may reveal dimensions of psychological experience that are difficult to capture through polarity labels alone [17,18]. This approach reinforces the need to frame health and well-being within the context of patients’ lived experiences and social environments.

We employed NLP techniques to analyze the interview data. NLP leverages computational methods to process and interpret human language, extracting valuable insights. Recent advancements in deep learning have significantly improved the accuracy of analyzing textual meanings and emotions. In this study, we employed NLP models such as BERT (Bidirectional Encoder Representations from Transformers) [19] and LUKE (Language Understanding with Knowledge-based Embeddings) [20] to analyze the interviews. The aim of this study was to develop and evaluate NLP-based emotion classification models for Japanese cancer survivor interview narratives using both 3-class sentiment polarity and Plutchik 8-emotion frameworks as complementary descriptive perspectives and to compare models fine-tuned on interview narratives with models transferred from a general emotion dataset.

## Methods

### Research Design

**Figure 1.**
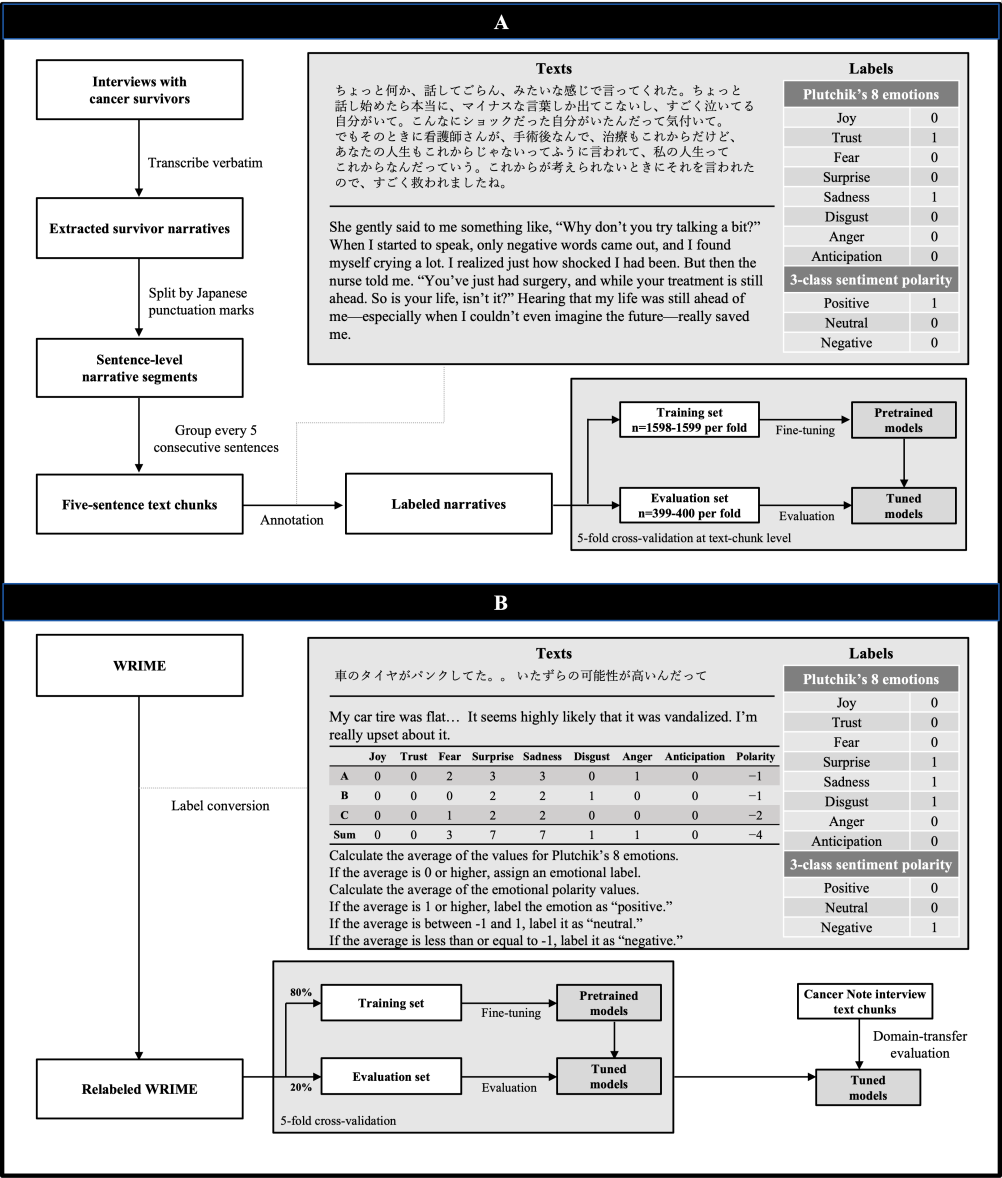
Data preparation and model evaluation procedure for (A) Japanese cancer survivor interview narratives and (B) WRIME (writers’ and readers’ intensities of emotion for their estimation) transfer analysis.

We analyzed Japanese interviews with cancer survivors, transcribed verbatim by the Cancer Note, Nonprofit Organization. Narratives from these interviewees were extracted and then segmented to create the target texts. The emotion labels were assigned according to predefined annotation guidelines. The emotions addressed in this study include categories based on sentiment polarity (positive, neutral, and negative) and 8 categories based on the Plutchik 8 basic emotions (joy, sadness, fear, trust, anticipation, anger, disgust, and surprise). Subsequently, 2 pretrained NLP models—BERT and LUKE—were fine-tuned using the labeled Cancer Note dataset, and classifiers were evaluated using 5-fold cross-validation at the text-chunk level. For comparison, BERT- and LUKE-based models were also fine-tuned on WRIME (writers’ and readers’ intensities of emotion for their estimation) and evaluated on the Cancer Note interview texts as a domain-transfer analysis (Figure 1).

As shown in Figure 1A, the cancer survivor interview texts were processed to facilitate the emotion analysis. Annotation was conducted such that 3-class sentiment labels were assigned in a multiclass format, wherein only 1 label could be selected. Conversely, the 8-emotion labels were assigned in a multilabel format, allowing multiple labels to be assigned simultaneously. The annotated Cancer Note dataset was evaluated using 5-fold cross-validation at the text-chunk level. In each fold, approximately 80% of the 1998 text chunks were used for model training and the remaining 20% were used for evaluation, corresponding to 1598 to 1599 training chunks and 399 to 400 evaluation chunks per fold.

As shown in Figure 1B, the WRIME dataset [21] was adjusted to align with the analytical methods used in this study. WRIME includes emotions assigned to each text by the author as well as by 3 readers. In this study, we used the readers’ emotion scores to provide a more objective perspective. The average emotion scores of the 3 readers were calculated, and emotion labels were assigned in the same manner as in the cancer survivor interview texts. Five WRIME-trained models were obtained from the 5 WRIME cross-validation folds. Each model was applied to the Cancer Note interview texts, and the logits from the 5 models were averaged to evaluate transfer performance from WRIME to the Cancer Note interview domain.

### Dataset

The dataset comprised verbatim transcription texts from cancer survivor interviews, typically lasting 90 minutes, conducted by the Cancer Note, Nonprofit Organization, titled “Cancer Note” [22]. These interviews were originally collected and published by Cancer Note for the purpose of sharing cancer survivors’ experiences and are publicly accessible on the Cancer Note’s website [22], as well as on its official YouTube channel [23]. They were not developed specifically for this study.

The interviewees were mainly cancer survivors who were either currently undergoing or had previously received cancer treatment. The Cancer Note interviews encompassed a broad spectrum of topics related to patients’ daily lives, including social relationships, financial circumstances, romantic involvement, and marital status. We randomly selected 15 narratives from 64 interviews conducted between March 2017 and March 2020. Because this study was designed as an exploratory model development and evaluation study using existing publicly available survivor narratives, the sample size was determined pragmatically based on transcript availability and the feasibility of manual multilabel emotion annotation, rather than by a statistical power calculation.

To facilitate the development of the sentiment classification model, a preprocessing protocol was implemented for the transcripts. We primarily extracted utterances made by cancer survivors themselves and removed elements that were unlikely to convey meaningful emotional content, such as nodding, back-channel responses, short perfunctory responses, interviewer prompts, and nonsubstantive conversational fillers. Closing remarks at the end of each interview were also removed to avoid conflating data from different interviews or including formulaic expressions unrelated to the main narrative content.

The remaining narratives were segmented at Japanese punctuation marks into sentence-level units and then grouped into blocks of 5 consecutive sentences within the same interview. This 5-sentence grouping was chosen after discussion among the annotators to balance 3 considerations: preserving sufficient local context for emotion interpretation, remaining within the 512-token input limit of the pretrained models, and maintaining feasible annotation units. We considered single-sentence segmentation and longer paragraph-level segmentation as alternatives. However, single-sentence units were considered more likely to lose contextual cues needed for emotion interpretation, whereas longer units could exceed the model input limit and increase annotation difficulty.

We acknowledge that this rule-based segmentation may still disrupt broader narrative coherence or emotional flow across longer interview passages. Therefore, the resulting chunks should be interpreted as local narrative segments rather than complete representations of each participant’s emotional trajectory. This preprocessing procedure produced a corpus of 1998 texts for analysis. We did not empirically compare alternative segmentation strategies in this study because changing the segmentation unit would also change the annotation unit and would likely require additional annotation.

### Annotation

Two annotators (S Hisamura and KS) annotated 1998 texts. For each text, one of 3-sentiment polarities (“positive,” “neutral,” or “negative”) and the Plutchik 8 basic emotions were assigned. The 8-emotion labels were assigned in a multilabel format, allowing multiple labels per text. In the 3-class polarity task, “neutral” was defined as the absence of a clearly dominant positive or negative evaluative direction within the text, rather than the complete absence of emotional content. Therefore, texts containing mixed, ambiguous, or weak emotional cues could be labeled as neutral in the polarity task when neither positive nor negative polarity was dominant.

For the descriptive comparison between the 3-class polarity labels and the Plutchik 8-emotion labels, joy and trust were treated as positive emotions, whereas sadness, fear, disgust, and anger were treated as negative emotions. Anticipation was treated as positive in this study because it was operationalized as forward-looking expectation, hope, or determination in the context of survivorship narratives. Surprise was treated as neutral because it does not have a fixed valence and may be associated with either positive or negative interpretations depending on context. This mapping was used as an operational framework for descriptive comparison, rather than as a claim that these emotions always have fixed valence across all contexts.

Because emotion annotation in the narrative text involves substantial interpretive complexity, we conducted a preliminary calibration step before the main annotation process. Specifically, 100 texts were randomly selected from the 1998 target texts as an overlapping subset for interrater agreement assessment. Both annotators independently annotated these 100 texts, and Cohen κ coefficients [24] were calculated based on this doubly annotated subset. Disagreements identified in this subset were reviewed through discussion and used to refine the annotation guidelines before the completion of the full annotation. This discussion was intended to calibrate the annotators’ interpretation of the guidelines rather than to perform consensus labeling for all 1998 texts.

After this calibration step, the full set of 1998 texts was annotated for model development, with S Hisamura annotating 1292 texts from 10 interviews and KS annotating 706 texts from 5 interviews. Final labels for the main analytic dataset were assigned according to the refined annotation guidelines. Interrater reliability was assessed using Cohen κ coefficient [24], which quantifies the degree of agreement between 2 raters, excluding the effect of chance agreement.

For comparative analysis, we used the WRIME dataset developed by Kajiwara and Nakashima [21]. WRIME consists of Japanese social media posts annotated with emotion labels from both the writer’s and readers’ perspectives [25]. In this study, we used the reader-assigned emotion scores and converted them to align with the 3-class sentiment polarity and the Plutchik 8-emotion classification framework used for the Cancer Note interview texts. Models were fine-tuned on WRIME and then evaluated on the Cancer Note interview texts to examine transfer performance from a general-purpose emotion dataset to the cancer survivor interview domain. Specifically, 5 WRIME-trained models were obtained from the 5 WRIME cross-validation folds, and each model was applied to the Cancer Note interview texts. The logits from the 5 models were averaged for each Cancer Note text chunk to generate the final transfer predictions. This comparison was intended as a domain-transfer reference rather than as a direct comparison between fully equivalent datasets, because WRIME and the Cancer Note interview narratives differ in text source, narrative context, and annotation process.

### Tasks and Metrics

For each interview narrative text, we conducted 2 classification tasks: a multiclass classification to categorize the text as “positive,” “neutral,” or “negative” and a multilabel classification to identify the presence of the Plutchik 8 emotions. We also calculated the co-occurrence of emotion labels and analyzed patterns of simultaneous emotion occurrences, defined as instances where 2 different emotions were simultaneously observed in the text. These 2 tasks were conducted in parallel as complementary descriptive analyses rather than as a hierarchical or multitask framework in which the polarity classification was used to improve multilabel emotion detection.

We used precision, recall, and *F*_1_-score for each label to evaluate class-specific performance. Macroaveraged precision, recall, and *F*_1_-score were calculated to summarize performance across labels while giving equal weight to each class. Micro-*F*_1_ was calculated as an overall performance metric for the 3-class sentiment polarity task. For the 8-emotion multilabel classification task, Hamming loss was calculated as an additional metric to assess label-level prediction errors because complete-label matching can be overly strict and insensitive to partial correctness in multilabel classification. Detailed label-wise precision, recall, and *F*_1_-score values are provided in Multimedia Appendix 1.

For the models trained on the Cancer Note texts, performance was evaluated using 5-fold cross-validation at the text-chunk level. In each fold, approximately 80% of the 1998 text chunks were used for training and the remaining 20% were used for evaluation, corresponding to 1598 to 1599 training chunks and 399 to 400 evaluation chunks per fold. The reported performance values were aggregated across the 5 folds. Thus, the 4:1 ratio refers to the training/evaluation ratio within each cross-validation fold, not to a separate hold-out test split.

For the WRIME-trained transfer models, 5 models were obtained from the 5 WRIME cross-validation folds. Each WRIME-trained model was applied to the full Cancer Note interview dataset, and logits were saved for each model. For the final transfer evaluation, logits from the 5 WRIME-trained models were averaged for each Cancer Note text chunk, and final predicted labels were assigned based on the resulting output probabilities. This approach was used to evaluate transfer performance from WRIME to the Cancer Note interview domain.

For the main performance metrics, 95% CIs were estimated using bootstrap resampling with 1000 iterations. For each bootstrap sample, text chunks were resampled with replacement, and the corresponding true labels and model predictions were used to recalculate the performance metrics. The 2.5th and 97.5th percentiles of the bootstrap distribution were used as the lower and upper bounds of the 95% CI.

The dataset exhibited substantial label imbalance, particularly in the 8-emotion classification task. In the present exploratory study, we did not apply explicit imbalance-mitigation strategies such as focal loss, class weighting, oversampling, synthetic data augmentation, or label-specific threshold tuning during training. This decision was made because our primary aim was to first evaluate baseline performance under the original label distribution of the interview narratives using a consistent evaluation framework across models. We therefore interpreted the results with particular attention to macroaveraged and label-wise performance, especially for low-frequency labels.

### Model Architecture

NLP models such as BERT typically undergo a 2-phase training process. Initially, they are pretrained on extensive corpora, such as Wikipedia, to develop a general language model independent of specific tasks (pretrained model). Subsequently, the models are fine-tuned using a limited set of labeled data to specialize in the target task.

We developed an emotion classifier using the Japanese pretrained BERT model [26] (cl-tohoku/bert-base-Japanese), which was developed by the Inui-Suzuki Laboratory at Tohoku University, in conjunction with the Japanese version of the LUKE model [27] (studio-ousia/luke-japanese-base), a knowledge-enhanced language model developed by Studio Ousia.

The text was tokenized via morphological analysis. The special tokens “[CLS]” and “[SEP]” were added at the beginning of the text, between sentences, and at the conclusion of the text to serve as model inputs. We did not systematically assess morphological tokenization errors in the colloquial speech transcripts. Because the transcripts were derived from spoken interviews and included colloquial expressions, tokenization errors may have affected model inputs and classification performance.

The output indicates positive, neutral, or negative emotions in the 3-class sentiment polarity classification task and shows the presence or absence of each emotion in the 8-emotion multilabel classification task. LUKE, such as BERT, employs tokenized text as input but additionally identifies entities within the text, utilizing them as supplementary model inputs. Fine-tuning was performed using an AdamW optimizer with a learning rate of 5e-5. The training batch size was 16 for both tasks. Models for the 3-class sentiment polarity task were fine-tuned for 3 epochs. For the 8-emotion multilabel classification task, models trained on the Cancer Note interview narratives were fine-tuned for 10 epochs, whereas models trained on WRIME were fine-tuned for 3 epochs. These settings were prespecified and were not systematically tuned in this exploratory study.

For the multiclass sentiment polarity classification task, we employed the 768-dimensional vector corresponding to [CLS] from the output vectors of the pretrained models as input to the classification layer. This layer comprises a fully connected layer that outputs logits representing the probability of each class. The softmax activation function was applied to determine the final label output. CrossEntropyLoss was employed as the loss function.

For the multilabel classification task based on the Plutchik 8 emotions, we similarly utilized the 768-dimensional vector corresponding to [CLS] from the output vectors of the pretrained models as the input to the classification layer. This layer comprises a fully connected layer that outputs logits representing the probabilities of the presence of each of the 8 emotions. The sigmoid activation function was applied, with the threshold set to 0.5. All labels exceeding this threshold were considered final output labels. BCEWithLogitsLoss was employed as the loss function.

Utilizing these 2 emotion classification models, we developed classifiers based on narratives from cancer survivor interviews and a comparative WRIME dataset using pretrained BERT and LUKE models.

### Ethical Considerations

This study was approved by the Ethics Committee of Keio University Faculty of Pharmacy (approval number 230127‐1). In accordance with the “Ethical Guidelines for Life Science and Medical Research Involving Human Subjects” and the Declaration of Helsinki, the committee determined that no new informed consent was required because the narratives had been shared by survivors through Cancer Note under a participation agreement that permits the use of their interview transcripts for research and publication purposes. We nonetheless recognized that the original sharing was intended to communicate survivor experiences to the public rather than to support NLP model development and that the specific application of language models for emotion classification may not have been anticipated by individual participants at the time of original sharing. To address this gap between the original purpose of sharing and the present analytical use, we treated the narratives as secondary-use data and applied additional safeguards beyond the original agreement: an opt-out announcement was posted on the Cancer Note website before the analysis began, and no participant requested exclusion; transcripts were anonymized prior to model training so that classifier outputs could not be linked to identifiable individuals. To respect participants’ likely expectation that sharing their experiences would contribute to a broader understanding of cancer survivorship, the classifiers were developed as research tools to characterize patterns across narratives rather than to label, profile, or make decisions about any individual participant.

## Results

### Dataset Statistics

The average word count per narrative text chunk in the cancer survivor interviews was 136.9 (SD 71.1), with a median of 124.0 (range 24-636) words. Table 1 presents the κ coefficients and the number of labels assigned in the annotation test. Regarding the κ coefficient, the highest agreement among annotators in the 3-sentiment classification was for “negative,” while “positive” had the lowest agreement. In the 8-emotion classification, “fear” showed the highest agreement, followed by “joy” and “anger;” “anticipation” exhibited the lowest agreement.

Concerning the number of labels assigned, “neutral” was the most frequently assigned label in the 3-sentiment classification, and “positive” was the least frequent. In the 8-emotion classification, “trust” had the most labels, whereas “anger” had the fewest. The frequency of the most commonly assigned label among the 3 sentiments and 8 emotions was 3.47 times and 8.10 times higher, respectively, than that of the least commonly assigned label, highlighting a marked imbalance in label distribution. Multimedia Appendix 2 provides specific examples of text assigned to each emotion label in both classifications.

**Table 1.** Cohen κ coefficients and label distributions in 1998 Japanese cancer survivor interview narrative text chunks.

Labels	Cohen κ	Texts, n (%)
Three sentiments based on emotional polarity		
Positive	0.36	343 (17.2)
Neutral	0.40	1191 (59.6)
Negative	0.57	464 (23.2)
The Plutchik 8 emotions		
Joy	0.51	99 (5.0)
Trust	0.37	324 (16.2)
Fear	0.54	198 (9.9)
Surprise	0.38	93 (4.7)
Sadness	0.42	268 (13.4)
Disgust	0.37	240 (12.0)
Anger	0.49	40 (2.0)
Anticipation	0.32	199 (10.0)

**Figure 2.**
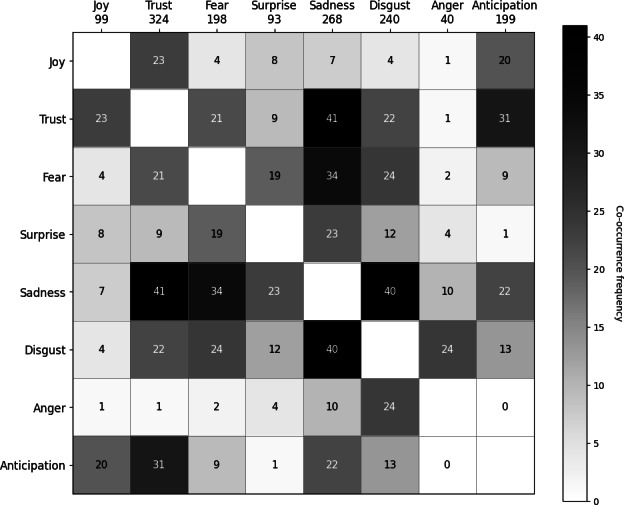
Co-occurrence matrix of the Plutchik 8 emotions in 1998 text chunks from Japanese cancer survivor interview narratives.

Table 2 displays the number of the 8-emotion labels assigned to the texts labeled with each of the 3-class sentiment labels. Texts labeled “positive” or “negative” often received many “positive” or “negative” labels among the 8 emotions. In the emotion co-occurrence analysis, “sadness” and “trust” co-occurred most frequently, whereas “anger” and “anticipation” showed the lowest co-occurrence frequency (Figure 2).

Numbers below each emotion label indicate the total number of text chunks assigned to that emotion label. Each off-diagonal cell represents the number of text chunks in which 2 distinct emotion labels were assigned simultaneously. Cell values and the color scale indicate co-occurrence frequencies; darker shading indicates higher co-occurrence. Diagonal cells were left blank because they do not represent co-occurrence between distinct emotions.

**Table 2.** Co-occurrence of the Plutchik 8-emotion labels with 3 sentiment polarity labels in 1998 Japanese cancer survivor interview text chunks.

Labels	Positive (n=343)	Neutral (n=1191)	Negative (n=464)
Joy	74	19	6
Trust	177	113	34
Fear	12	50	136
Surprise	9	40	44
Sadness	22	42	204
Disgust	11	35	194
Anger	0	3	37
Anticipation	137	48	14

### Model Performance

As shown in Table 3, in the 3-sentiment polarity classification task, models fine-tuned on cancer survivor interview narratives showed higher overall performance than WRIME-trained transfer models. Interview Text-BERT achieved the highest micro-*F*_1_ score of 0.696 (95% CI 0.676‐0.716), whereas Interview Text-LUKE achieved the highest macro-*F*_1_ score of 0.660 (95% CI 0.639‐0.682). WRIME-trained models showed lower transfer performance on the Cancer Note interview texts, with micro-*F*_1_ scores of 0.559 (95% CI 0.537‐0.581) for WRIME-BERT and 0.612 (95% CI 0.591‐0.634) for WRIME-LUKE.

Detailed label-wise performance values are provided in Multimedia Appendix 1. In brief, the *F*_1_-score for “neutral” was relatively high in the models trained on cancer survivor interview narratives, whereas the *F*_1_-scores for “positive” and “negative” were lower. This tendency was less apparent in the WRIME-trained transfer models.

**Table 3.** Three-class sentiment polarity classification^a^.

Model	Macro-precision, mean (95% CI)	Macro-recall, mean (95% CI)	Macro*-F*_1_, mean (95% CI)	Micro*-F*_1_, mean (95% CI)
Interview Text-BERT^b^	0.655 (0.632‐0.682)	0.645 (0.622‐0.669)	0.650 (0.628‐0.674)	0.696 (0.676‐0.716)
Interview Text-LUKE^c^	0.646 (0.624‐0.669)	0.683 (0.661‐0.705)	0.660 (0.639‐0.682)	0.688 (0.668‐0.708)
WRIME-BERT^d^	0.550 (0.527‐0.574)	0.592 (0.569‐0.616)	0.547 (0.524‐0.569)	0.559 (0.537‐0.581)
WRIME-LUKE^e^	0.598 (0.576‐0.619)	0.673 (0.650‐0.696)	0.608 (0.585‐0.630)	0.612 (0.591‐0.634)

a Values are shown as mean performance with 95% CIs estimated using bootstrap resampling with 1000 iterations.

b Interview Text-BERT: A BERT (Bidirectional Encoder Representations from Transformers)–based model fine-tuned on cancer survivor interview narratives.

c Interview Text-LUKE: A LUKE (Language Understanding with Knowledge-based Embeddings)–based model fine-tuned on cancer survivor interview narratives.

d WRIME-BERT: BERT-based model fine-tuned on WRIME (writers’ and readers’ intensities of emotion for their estimation) and evaluated on cancer survivor interview narratives.

e WRIME-LUKE: LUKE-based model fine-tuned on WRIME and evaluated on cancer survivor interview narratives.

As shown in Table 4, in the 8-emotion multilabel classification task, Interview Text-LUKE achieved the highest overall performance among the 4 models, with a macro-*F*_1_ score of 0.427 (95% CI 0.398‐0.453) and the lowest Hamming loss of 0.078 (95% CI 0.073‐0.082). Interview Text-BERT showed a macro-*F*_1_-score of 0.342 (95% CI 0.317‐0.363) and a Hamming loss of 0.082 (95% CI 0.078‐0.087). WRIME-trained models showed lower transfer performance, with macro-*F*_1_ scores of 0.310 (95% CI 0.286‐0.333) for WRIME-BERT and 0.341 (95% CI 0.319‐0.363) for WRIME-LUKE. Their Hamming losses were also higher than those of the interview-trained models, at 0.217 (95% CI 0.212‐0.223) for WRIME-BERT and 0.192 (95% CI 0.187‐0.198) for WRIME-LUKE.

Detailed label-wise results for the 8-emotion classification task are provided in Multimedia Appendix 1. In brief, *F*_1_-scores were relatively higher for emotions such as trust, anticipation, disgust, sadness, and fear, whereas performance was lower for anger, joy, and surprise. Thus, within this exploratory setting, models fine-tuned on cancer survivor interview narratives generally showed better performance than WRIME-trained transfer models, although the best-performing architecture differed depending on the task and evaluation metric.

The overall performance of BERT- and LUKE-based models in the sentiment polarity and the Plutchik 8-emotion classification tasks using Japanese cancer survivor interview narratives is shown in Tables3  4.

**Table 4.** Eight-emotion multilabel classification based on the Plutchik emotion framework^a^.

Model	Macro-precision, mean (95% CI)	Macro-recall, mean (95% CI)	Macro-*F*_1_, mean (95% CI)	Hamming loss, mean (95% CI)
Interview Text-BERT^b^	0.465 (0.426‐0.501)	0.277 (0.257‐0.297)	0.342 (0.317‐0.363)	0.082 (0.078‐0.087)
Interview Text-LUKE^c^	0.577 (0.491‐0.631)	0.369 (0.347‐0.393)	0.427 (0.398‐0.453)	0.078 (0.073‐0.082)
WRIME-BERT^d^	0.318 (0.274‐0.360)	0.565 (0.540‐0.592)	0.310 (0.286‐0.333)	0.217 (0.212‐0.223)
WRIME-LUKE^e^	0.313 (0.279‐0.350)	0.598 (0.574‐0.625)	0.341 (0.319‐0.363)	0.192 (0.187‐0.198)

a Values are shown as mean performance with 95% CIs estimated using bootstrap resampling with 1000 iterations. Hamming loss represents the fraction of incorrectly predicted labels in the multilabel classification task.

b Interview Text-BERT: BERT (Bidirectional Encoder Representations from Transformers)-based model fine-tuned on cancer survivor interview narratives.

c Interview Text-LUKE: LUKE (Language Understanding with Knowledge-based Embeddings)-based model fine-tuned on cancer survivor interview narratives.

d WRIME-BERT: BERT-based model fine-tuned on WRIME (writers’ and readers’ intensities of emotion for their estimation) and evaluated on cancer survivor interview narratives.

e WRIME-LUKE: LUKE-based model fine-tuned on WRIME and evaluated on cancer survivor interview narratives.

## Discussion

### Principal Findings

In relation to the study’s aim of developing and evaluating NLP-based emotion classification models for cancer survivor interview narratives, this study found that models fine-tuned on interview narratives generally achieved higher performance than WRIME-trained transfer models within this exploratory setting. In particular, LUKE showed the strongest performance in the 8-emotion multilabel classification task, whereas the best-performing architecture in the 3-class sentiment polarity task differed depending on the evaluation metric. The analysis also showed that polarity labels and Plutchik 8-emotion labels provided complementary descriptive perspectives on coexisting emotions in survivorship narratives.

Our analysis of the Cancer Note interview narratives suggested that survivors’ experiences often involve coexisting and sometimes contrasting emotions across the cancer trajectory. For example, narratives around diagnosis and treatment frequently included negative emotions such as sadness and disgust, while narratives about life after treatment also included positive emotions such as anticipation and trust in health care professionals and family members. These findings highlight the complexity of cancer survivors’ psychological experiences and suggest that future narrative-based support should acknowledge both positive and negative emotional elements within the same narrative.

In this sentiment analysis, we developed emotion classifiers by fine-tuning pretrained LUKE and BERT models using interview data from cancer survivors. Overall, models fine-tuned on the interview narratives achieved higher performance than models fine-tuned on the general emotion dataset WRIME when evaluated on the interview domain. In the 3-class sentiment polarity classification task, Interview Text-BERT achieved the highest micro-*F*_1_, whereas Interview Text-LUKE achieved the highest macro-*F*_1_. In the 8-emotion multilabel classification task, Interview Text-LUKE achieved the highest macro-*F*_1_ and the lowest Hamming loss among the 4 models. The lower transfer performance of WRIME-trained models may reflect domain-specific language in survivorship narratives, including cancer-related expressions that are less prevalent in diverse social media posts. However, this comparison should be interpreted cautiously because WRIME and the Cancer Note interview narratives differ not only in domain but also in annotation construct and narrative context. WRIME consists of social media posts with reader-assigned emotions, whereas the present dataset consists of retrospective cancer survivor interview narratives annotated according to a study-specific framework. Therefore, the observed performance differences may reflect a combination of domain specificity, differences in narrative style, text length, and annotation philosophy rather than domain specificity alone. In addition, LUKE tended to show stronger performance than BERT in several macroaveraged and multilabel metrics, although the best-performing architecture differed depending on the task and evaluation metrics. One possible explanation is LUKE’s entity-aware pretraining, which may better represent clinical entities such as disease names and anticancer drug names and thereby support classification of narrative passages that refer to treatments, clinicians, and disease-related experiences. Nevertheless, this explanation is speculative and should be examined in future analyses.

Prior work has shown that NLP can extract clinically relevant psychosocial content from patient-generated narratives in oncology, including domain-adapted BERT approaches for identifying concerns or worries in breast cancer narratives from interviews and blogs [15,16]. More broadly, a systematic review of sentiment analysis in health and well-being has highlighted the rapid growth of computational approaches for characterizing subjective experiences from the free text, while also noting challenges related to domain specificity and interpretability [17]. However, most cancer-related NLP studies have focused on polarity-based sentiment classification or on text sources such as social media posts, forums, and other online narratives rather than long-form retrospective interview transcripts [17,28-30]. For example, recent work on cancer narratives from Reddit has emphasized the linguistic complexity of patient and caregiver narratives and the difficulty of reliably performing sentiment or emotion analysis on longer, context-dependent texts [28]. Similarly, studies mining cancer-related social media in different languages and settings have reported that emotion and symptom expressions vary with platform and population, reinforcing the likelihood of domain shift when models are trained outside the target narrative domain [29,30].

Against this background, our study contributes by focusing on Japanese cancer survivor interview narratives and by evaluating a dual descriptive framework that combines 3-class polarity labels with a multidimensional emotion framework (the Plutchik 8 emotions). While the use of Plutchik-based labels has been explored in NLP research outside oncology and shown to provide a structured basis for emotion classification [31], comparatively fewer studies have examined multidimensional emotion labeling in survivor interview transcripts and assessed whether models trained on general-purpose emotion datasets can transfer to retrospective survivorship narratives. Our findings extend prior cancer narrative NLP work [15,16] by (1) quantifying the performance gap between interview-trained models and models fine-tuned on a general emotion dataset (WRIME) in a domain-transfer evaluation and (2) illustrating how polarity labels and the Plutchik emotion labels can provide complementary perspectives on coexisting emotions in survivorship narratives. In our analysis, the 2 tasks were designed as complementary descriptive analyses rather than as a hierarchical framework in which polarity classification was expected to improve multilabel emotion detection. While emotions such as “trust” and “anticipation” generally aligned with positive sentiment and “sadness” and “disgust” with negative sentiment, notable exceptions were observed. In particular, “trust” co-occurred frequently with “sadness,” illustrating how positive and negative feelings can coexist within the same narrative and challenging the simplicity of polarity-based categorization. This pattern is consistent with psychosocial oncology literature describing mixed and dynamic emotional experiences in survivorship. Together, these results support the view that domain-specific narrative data and multidimensional emotion frameworks may be necessary to characterize context-dependent emotions in survivorship interviews, while also underscoring the need for larger and more diverse datasets and further validation before clinical translation.

Moreover, this analysis showed that some texts labeled as “positive” or “negative” within the 3-class sentiment polarity framework contained subtle emotional elements not fully captured by either polarity or specific emotions. Co-occurrences of contrasting emotions such as “trust” and “sadness” illustrate this complexity, suggesting that cancer survivors’ emotional experiences are dynamic and multifaceted. In the annotation process, “trust” emerged as the most frequent label, often associated with family support and confidence in health care professionals. Texts labeled with “anticipation” expressed determination and optimism for the future. “Sadness” was predominantly assigned to narratives about the moment of diagnosis, frequently co-occurring with “surprise.” “Disgust” and “fear” were commonly linked to descriptions of pain and treatment-related hardship, with “fear” often reflecting concerns about future physical conditions. Notably, the prevalence of “disgust” contrasted with previous research on patients with cancer in clinical trials [32], suggesting that this emotion may be distinctive to cancer survivors’ narratives.

Conventionally, “joy” and “trust” are categorized as positive emotions, while “fear,” “sadness,” “disgust,” and “anger” are categorized as negative emotions. In this study, “anticipation” was also classified as positive, while “surprise” was considered neutral. As shown in Table 2, “positive” texts were more often associated with “trust,” “anticipation,” or “joy,” whereas “negative” texts frequently included “fear,” “sadness,” or “disgust.” Texts labeled as “neutral” were the most common in the 3-class sentiment polarity framework but were less frequently associated with Plutchik 8-emotion labels than positive or negative texts.

The error analysis suggested that some misclassifications involved confusion between “positive” and “negative” labels. These errors likely stemmed from the coexistence of “positive” and “negative” emotional elements within the same text, complicating classification. In the 8-emotion framework, emotions such as “joy,” “surprise,” and “anger” demonstrated poorer performance, likely due to their varied expressions and the imbalance in label distribution, as these emotions were assigned to relatively few text chunks and were expressed in varied contexts.

The poor performance observed for rare labels such as anger should also be interpreted in light of the marked class imbalance in the dataset. Because this study was designed as an exploratory evaluation of models trained under the original interview-derived label distribution, we did not apply explicit imbalance-mitigation procedures such as focal loss, class weighting, oversampling, synthetic augmentation, or label-specific threshold tuning. Accordingly, the results for low-frequency emotions should be interpreted cautiously. The comparative performance across models can still be interpreted as an exploratory within-study comparison because all models were evaluated on the same Cancer Note label distribution and within a consistent evaluation framework. However, these comparisons should not be taken as the definitive evidence of robust performance for rare emotions.

### Limitations

This study had several limitations related to the retrospective nature of the data, annotation uncertainty, preprocessing, model evaluation, and generalizability.

First, the retrospective nature of cancer survivors’ narratives may introduce recall bias and temporal distortion. Emotions expressed during interviews may not fully reflect the emotions experienced at the time of the events but may instead reflect reconstructed emotional meaning shaped by subsequent experiences, coping, social context, and the interview setting. Therefore, models trained on these retrospective narratives should not be assumed to generalize directly to real-time or prospective clinical monitoring of patients’ emotional states.

Second, the annotation process involved inherent uncertainty. Interannotator agreement showed κ coefficients ranging from 0.30 to 0.60, indicating fair-to-moderate agreement, particularly for nuanced and context-dependent emotions such as anticipation and trust. Although disagreements in the overlapping annotation subset were discussed and used to refine the annotation guidelines, full adjudication or consensus labeling of all 1998 texts was not performed. We also did not conduct sensitivity analyses using alternative labels or repeated annotations. Future studies should consider methods to improve multilabel emotion annotation reliability, such as refining annotation criteria, using score-based annotation and applying thresholding procedures for emotion labels [33]. Therefore, the reported metrics should be interpreted as performance against a practically constructed but imperfect reference label set rather than a definitive ground truth.

Third, preprocessing and evaluation choices may have affected model performance. Five-sentence segmentation was used to preserve local context while remaining within model input constraints, but this rule-based approach may have interrupted longer emotional sequences or broader narrative flow. We did not conduct an empirical ablation analysis comparing alternative segmentation strategies. In addition, cross-validation was performed at the text-chunk level rather than at the interview level. Therefore, although identical text chunks did not appear in both training and evaluation folds, different chunks from the same interview may have appeared in both sets, potentially leading to optimistic performance estimates. Interview-level cross-validation was considered but not adopted because, given only 15 interviews and the marked class imbalance (eg, anger 2.0%), each interview-level evaluation fold would contain very few or even zero instances of rare emotion labels, producing unstable or undefined per-label metrics. In addition, because individual interviews tend to focus on different topics, treatment trajectories, and vocabulary, interview-level splits would shift the evaluation toward testing out-of-distribution generalization rather than the within-domain feasibility that was the primary aim of the present exploratory study. Dedicated interview-level or external validation using a larger and more diverse cohort therefore remains an important direction for future work.

Fourth, the imbalanced distribution of emotion labels likely affected performance, particularly for rare emotions. The least frequent label, such as anger, appeared only 40 times, and some evaluation folds may have contained very few anger-labeled texts. Because we did not apply imbalance-mitigation strategies such as focal loss, class weighting, oversampling, synthetic augmentation, or label-specific threshold tuning, the low performance for rare emotions should be interpreted cautiously. Model comparisons should therefore be regarded as exploratory rather than as the definitive evidence of robust rare-label detection.

Finally, generalizability may be limited because the dataset was derived from only 15 interviews from a single platform and a single language context. Although the analysis included 1998 text chunks, these chunks originated from a limited number of source narratives and may not represent fully independent or sufficiently diverse samples. Because Cancer Note is designed for public sharing of survivorship experiences, its narratives may be more reflective, structured, or socially oriented than clinical notes, real-time patient-reported outcomes, or informal conversations. These platform-specific storytelling styles could limit transferability to clinical notes, real-time patient-reported outcomes, or informal conversations. Even when using pretrained architectures such as BERT and LUKE for fine-tuning, this limited diversity may increase the risk of overfitting to dataset-specific narrative patterns. The comparison with WRIME should likewise be interpreted cautiously because the datasets differ in text source, annotation perspective, and narrative context. Moreover, the dual framework was primarily descriptive, and we did not test whether polarity labels improved multilabel emotion detection. Future studies should evaluate these approaches using larger, more diverse, prospectively collected, and externally validated datasets, with improved annotation procedures, alternative segmentation strategies, interview-level validation, and imbalance-aware training methods.

### Clinical Considerations

This study advances efforts to incorporate patients’ emotional experiences into cancer care by demonstrating that nuanced emotional patterns can be extracted from cancer survivors’ narratives. By revealing co-occurring emotions and their relationships, this approach may contribute to a more contextual understanding of the psychological dynamics that affect patient well-being.

The findings also emphasize the importance of contextualizing patients’ emotions within their social and environmental settings, aligning with broader health models that prioritize patient-centered care. However, given the exploratory nature of this study and the modest performance of the 8-emotion classifier, the developed emotion classification models should be viewed as preliminary research tools rather than clinically actionable systems. The present results should not be interpreted as establishing clinically meaningful decision-support thresholds, and the models should not be used for screening, triage, or clinical decision-making without further validation. Because the present models were trained on retrospective interview narratives, their outputs should not be interpreted as indicators of real-time affect or as tools for prospective monitoring without validation on longitudinal or prospectively collected data.

Rather, the findings suggest that emotion classification may eventually serve as supplementary information for summarizing emotional patterns in narrative data under human oversight. Misclassification of distress-related emotions could lead to inappropriate reassurance, unnecessary concern, or missed opportunities for psychosocial support. Therefore, user-centered evaluation is needed to assess interpretability, safety, workflow integration, and appropriate use before any clinical implementation.

### Future Directions

To gain deeper insights into the social factors and emotional stressors affecting the well-being of patients with cancer [34], it may be beneficial to use the emotion classification model developed in this study alongside the patient concern model previously created by our team [15,16]. A combined approach could support more comprehensive, patient-centered interventions by linking emotional expressions with specific concerns and contextual factors, thereby addressing both individual emotional needs and broader social dynamics [35]. Future work should evaluate whether such integration improves interpretability and usefulness for clinical teams and support programs and whether it contributes to more targeted psychosocial support in practice [36].

Future research could further refine emotion classification models by tailoring them to specific cancer types, treatment modalities, or survivorship phases. Such specialization may enable more precise identification of emotional patterns and linguistic expressions that are characteristic of each clinical trajectory, potentially contributing to more targeted and responsive patient-centered care. In parallel, expanding the dataset and improving label balance—especially for low-frequency emotions such as anger—will be important to increasing robustness and reliability.

### Conclusions

To our knowledge, this study is among the early efforts to apply NLP models to analyze Japanese cancer survivor narratives with both a polarity-based sentiment analysis and a multidimensional emotion framework. By examining these perspectives, our approach captures both overarching trends and nuanced emotional states and illustrates that survivorship narratives may contain coexisting and contrasting emotions that are not fully represented by polarity alone. These findings suggest the feasibility of developing domain-specific emotion classifiers for survivorship narratives and provide a foundation for future work, including larger-scale validation, improved handling of label imbalance, and user-centered evaluation to clarify safe and meaningful clinical use.

Ultimately, advanced emotion analysis may contribute to future research on psychosocial support and patient-centered oncology care, provided that larger-scale validation, careful risk assessment, and user-centered implementation studies are conducted.

## Supplementary material

10.2196/94826 Multimedia Appendix 1 Label-wise performance of BERT- and LUKE-based emotion classification models.

10.2196/94826 Multimedia Appendix 2 Examples of a text labeled with both 3-class sentiment polarity and Plutchik 8-emotion labels.

## Abbreviations

NLP: natural language processing
BERT: Bidirectional Encoder Representations from Transformers
LUKE: Language Understanding with Knowledge-based Embeddings
WRIME: writers’ and readers’ intensities of emotion for their estimation
