# Supplementary material for: Emotion Classification in Japanese Cancer Survivor Interview Narratives Using Sentiment Polarity and Plutchik Emotion Frameworks: Model Development and Evaluation Study
Source: JMIR Form Res. 2026 Jun 30;10:e94826. doi: 10.2196/94826 (PMC13318080; doi:10.2196/94826)
Supplement: Multimedia Appendix 1 [file formative-v10-e94826-s001.docx]

**1. Label-wise performance of BERT- and LUKE-based emotion classification models**

This appendix presents detailed label-wise performance values for the 3-class sentiment polarity classification task and the 8-emotion multilabel classification task. The main manuscript reports overall model performance, including macro-averaged metrics, Micro-F1, and Hamming loss. This appendix provides label-wise precision, recall, and F1-score values for each model to support interpretation of class-specific performance. Values are shown as point estimates with 95% confidence intervals.

**Supplementary Table 1. Label-wise performance in the 3-class sentiment polarity classification task**

| **Label** | **Model** | **Support** | **Precision (95% CI)** | **Recall (95% CI)** | **F1 (95% CI)** |
| --- | --- | --- | --- | --- | --- |
| Positive | Interview Text-BERT | 343 | 0.625 (0.574–0.677) | 0.598 (0.547–0.646) | 0.611 (0.569–0.655) |
| Positive | Interview Text-LUKE | 343 | 0.566 (0.520–0.613) | 0.700 (0.652–0.747) | 0.626 (0.587–0.666) |
| Positive | WRIME-BERT | 343 | 0.506 (0.456–0.557) | 0.499 (0.446–0.548) | 0.502 (0.455–0.548) |
| Positive | WRIME-LUKE | 343 | 0.503 (0.460–0.547) | 0.685 (0.636–0.733) | 0.580 (0.538–0.622) |
| Neutral | Interview Text-BERT | 1191 | 0.757 (0.732–0.780) | 0.777 (0.754–0.800) | 0.767 (0.747–0.785) |
| Neutral | Interview Text-LUKE | 1191 | 0.786 (0.762–0.810) | 0.699 (0.673–0.726) | 0.740 (0.719–0.761) |
| Neutral | WRIME-BERT | 1191 | 0.710 (0.678–0.743) | 0.485 (0.459–0.513) | 0.577 (0.551–0.601) |
| Neutral | WRIME-LUKE | 1191 | 0.785 (0.754–0.816) | 0.507 (0.480–0.537) | 0.616 (0.590–0.642) |
| Negative | Interview Text-BERT | 464 | 0.583 (0.540–0.630) | 0.560 (0.517–0.608) | 0.571 (0.534–0.613) |
| Negative | Interview Text-LUKE | 464 | 0.586 (0.543–0.629) | 0.649 (0.604–0.692) | 0.616 (0.580–0.651) |
| Negative | WRIME-BERT | 464 | 0.435 (0.404–0.465) | 0.793 (0.757–0.828) | 0.562 (0.532–0.592) |
| Negative | WRIME-LUKE | 464 | 0.504 (0.470–0.534) | 0.828 (0.790–0.862) | 0.626 (0.596–0.653) |

**Supplementary Table 2. Label-wise performance in the 8-emotion multilabel classification task based on Plutchik’s emotion framework**

| **Label** | **Model** | **Support** | **Precision (95% CI)** | **Recall (95% CI)** | **F1 (95% CI)** |
| --- | --- | --- | --- | --- | --- |
| Joy | Interview Text-BERT | 99 | 0.382 (0.212–0.553) | 0.131 (0.066–0.198) | 0.195 (0.101–0.284) |
| Joy | Interview Text-LUKE | 99 | 0.439 (0.316–0.568) | 0.253 (0.173–0.333) | 0.321 (0.231–0.406) |
| Joy | WRIME-BERT | 99 | 0.188 (0.142–0.235) | 0.525 (0.427–0.624) | 0.277 (0.216–0.335) |
| Joy | WRIME-LUKE | 99 | 0.203 (0.165–0.243) | 0.758 (0.667–0.840) | 0.320 (0.269–0.375) |
| Trust | Interview Text-BERT | 324 | 0.639 (0.573–0.701) | 0.454 (0.400–0.506) | 0.531 (0.475–0.579) |
| Trust | Interview Text-LUKE | 324 | 0.664 (0.606–0.717) | 0.568 (0.513–0.622) | 0.612 (0.565–0.656) |
| Trust | WRIME-BERT | 324 | 0.710 (0.622–0.798) | 0.219 (0.177–0.266) | 0.335 (0.278–0.393) |
| Trust | WRIME-LUKE | 324 | 0.689 (0.612–0.766) | 0.287 (0.242–0.334) | 0.405 (0.350–0.459) |
| Fear | Interview Text-BERT | 198 | 0.558 (0.469–0.649) | 0.318 (0.255–0.385) | 0.405 (0.336–0.475) |
| Fear | Interview Text-LUKE | 198 | 0.621 (0.538–0.705) | 0.414 (0.347–0.480) | 0.497 (0.432–0.559) |
| Fear | WRIME-BERT | 198 | 0.178 (0.153–0.202) | 0.889 (0.845–0.931) | 0.297 (0.260–0.331) |
| Fear | WRIME-LUKE | 198 | 0.192 (0.165–0.217) | 0.848 (0.801–0.896) | 0.313 (0.275–0.347) |
| Surprise | Interview Text-BERT | 93 | 0.357 (0.192–0.546) | 0.108 (0.049–0.174) | 0.165 (0.080–0.256) |
| Surprise | Interview Text-LUKE | 93 | 0.319 (0.196–0.464) | 0.161 (0.094–0.245) | 0.214 (0.126–0.314) |
| Surprise | WRIME-BERT | 93 | 0.109 (0.087–0.133) | 0.774 (0.685–0.856) | 0.191 (0.156–0.229) |
| Surprise | WRIME-LUKE | 93 | 0.138 (0.110–0.171) | 0.742 (0.652–0.830) | 0.233 (0.190–0.279) |
| Sadness | Interview Text-BERT | 268 | 0.547 (0.479–0.612) | 0.433 (0.374–0.489) | 0.483 (0.425–0.538) |
| Sadness | Interview Text-LUKE | 268 | 0.577 (0.512–0.638) | 0.504 (0.443–0.565) | 0.538 (0.482–0.590) |
| Sadness | WRIME-BERT | 268 | 0.258 (0.227–0.290) | 0.735 (0.676–0.785) | 0.382 (0.344–0.419) |
| Sadness | WRIME-LUKE | 268 | 0.305 (0.271–0.339) | 0.765 (0.707–0.814) | 0.436 (0.396–0.475) |
| Disgust | Interview Text-BERT | 240 | 0.614 (0.528–0.697) | 0.358 (0.299–0.419) | 0.453 (0.389–0.514) |
| Disgust | Interview Text-LUKE | 240 | 0.650 (0.583–0.722) | 0.487 (0.428–0.549) | 0.557 (0.501–0.613) |
| Disgust | WRIME-BERT | 240 | 0.301 (0.257–0.349) | 0.508 (0.445–0.573) | 0.378 (0.329–0.425) |
| Disgust | WRIME-LUKE | 240 | 0.349 (0.301–0.400) | 0.512 (0.450–0.571) | 0.416 (0.366–0.465) |
| Anger | Interview Text-BERT | 40 | 0.000 (0.000–0.000) | 0.000 (0.000–0.000) | 0.000 (0.000–0.000) |
| Anger | Interview Text-LUKE | 40 | 0.750 (0.000–1.000) | 0.075 (0.000–0.171) | 0.136 (0.000–0.286) |
| Anger | WRIME-BERT | 40 | 0.500 (0.167–0.818) | 0.125 (0.032–0.233) | 0.200 (0.053–0.340) |
| Anger | WRIME-LUKE | 40 | 0.308 (0.077–0.583) | 0.100 (0.024–0.200) | 0.151 (0.037–0.282) |
| Anticipation | Interview Text-BERT | 199 | 0.624 (0.538–0.706) | 0.417 (0.347–0.489) | 0.500 (0.432–0.568) |
| Anticipation | Interview Text-LUKE | 199 | 0.594 (0.520–0.664) | 0.492 (0.423–0.559) | 0.538 (0.472–0.598) |
| Anticipation | WRIME-BERT | 199 | 0.296 (0.255–0.333) | 0.744 (0.683–0.803) | 0.423 (0.376–0.463) |
| Anticipation | WRIME-LUKE | 199 | 0.321 (0.278–0.363) | 0.769 (0.707–0.824) | 0.453 (0.405–0.499) |

Interview Text-BERT: BERT-based model fine-tuned on cancer survivor interview narratives.

Interview Text-LUKE: LUKE-based model fine-tuned on cancer survivor interview narratives.

WRIME-BERT: BERT-based model fine-tuned on WRIME and evaluated on cancer survivor interview narratives.

WRIME-LUKE: LUKE-based model fine-tuned on WRIME and evaluated on cancer survivor interview narratives.

CI: confidence interval.

Note. In the 3-class sentiment polarity task, support indicates the number of text chunks assigned to each mutually exclusive polarity label. In the 8-emotion multilabel task, support indicates the number of text chunks assigned to each emotion label; because multiple emotion labels could be assigned to a single text chunk, support values do not sum to 1,998.
