# Supplementary material for: Emotion Classification in Japanese Cancer Survivor Interview Narratives Using Sentiment Polarity and Plutchik Emotion Frameworks: Model Development and Evaluation Study
Source: JMIR Form Res. 2026 Jun 30;10:e94826. doi: 10.2196/94826 (PMC13318080; doi:10.2196/94826)
Supplement: Multimedia Appendix 2 [file formative-v10-e94826-s002.docx]

**2. Examples of a text labeled with both 3-class sentiment polarity and Plutchik's eight emotions**

| Original in Japanese | English translation | Labels |
| --- | --- | --- |
| 聞いた話だと。ありがとうございました。家族、友人、職場の人含め、本当に人のありがたさを知れた期間だったなと思うんです。僕はずっとサッカーやってて、中学校のサッカーチームの人たちとか、卒業してから本当に何年も会ってなかったりした人とかもいるんですけど、病気をしてみんなが集まって、いろんな全国から集まってお見舞いに来てくれたりとか、いろいろメッセージくれたりとか。本当に離れ離れだったんだけど、そういうところで自分は支えてもらったりとかっていうのが本当に気付けた。 | I heard about it. Thank you very much. Including family, friends, and coworkers, I feel it was a time when I truly came to appreciate people's kindness. I've been playing soccer all my life, and there are people from my middle school soccer team whom I hadn't seen for many years since graduation. But when I got sick, everyone gathered, coming from all over the country to visit me and send messages. Even though we had been apart, I realized that they were supporting me in such meaningful ways. | 3-class Sentiment Polarity |
|  |  | Positive |
|  |  | Plutchik’s Eight Emotions |
|  |  | Joy  Trust |
| ここで運命の出会いがあったわけです。私の主治医になる先生との出会いだったんですけど。どきどきしながら診察室で待っていたら、その先生がカラカラってカーテンを開けて、すごい優しい笑顔で、「大丈夫だよ」と。その笑顔と、優しさがあふれている言葉とか、全てにはあっと感動して。この先生にお任せしようって思いました。 | This was where I had a fateful encounter—with the doctor who would become my primary physician. As I nervously waited in the examination room, the doctor slid open the curtain with a gentle sound and, wearing an incredibly kind smile, said, "It's going to be okay." I was deeply moved by that smile and those words overflowing with kindness—by everything. I thought, "I'll entrust myself to this doctor." | 3-class Sentiment Polarity |
|  |  | Positive |
|  |  | Plutchik’s Eight Emotions |
|  |  | Anticipation  Trust |
| その言葉を言われた瞬間に、頭が真っ白になってしまって。目の前は真っ暗で。よくマンガでもありますけど、ハンマーとか、おっきな石ががあんって上から落ちてくるような感じの、そういう衝撃を受けました。先生がお話しされてるのも全く入ってこなくて。途中途中で、乳房全摘とか、そういう言葉だけがピックアップされて聞こえて、私、死ぬんだ、どうしようって、そんな感じでしたね。 | The moment I heard those words, my mind went completely blank, and everything went dark in front of me. It felt like something out of a manga, as if a hammer or a giant rock had fallen on me from above—that's how shocked I was. I couldn’t take in anything the doctor was saying; only certain words, like "mastectomy," stood out and stuck in my mind. I thought, "I’m going to die. What should I do?" That’s how I felt. | 3-class Sentiment Polarity |
|  |  | Neutral |
|  |  | Plutchik’s Eight Emotions |
|  |  | Sadness  Disgust  Surprise  Fear |
| また言ってるよみたいな顔してますけれど。本当にどうしたらいいのっていうと、だんだん心がすさんでいって。結構、看護師さんとかに当たっちゃうんですよね。ちょっとカーテンの締め方がね、ちょっと隙間が開いてるとね、すごくイラっとしたりとかして。駄目、ここでけんかするとまた入院する確率高いんだから、その後ちょっと関係がまずくなっちゃうから。 | They look at me like, 'Here we go again.' But when you truly don't know what to do, your heart gradually becomes weary. I end up taking it out on the nurses quite a bit. If the way they close the curtains leaves a slight gap, I get extremely irritated. I know I shouldn't; if I start arguing here, the chances of being hospitalized again are high, and our relationship will become strained afterward. | 3-class Sentiment Polarity |
|  |  | Negative |
|  |  | Plutchik’s Eight Emotions |
|  |  | Anger  Disgust |
